# Improving the biomedical research literature: insights from authors’ editors can help journal editors define and refine their core competencies

**DOI:** 10.12688/f1000research.13760.2

**Published:** 2018-02-21

**Authors:** Valerie Matarese, Karen Shashok

**Affiliations:** 1 Via Roma 10, I-31020 Vidor (TV), Italy; 2 C./ Compositor Ruiz Aznar 12, 2-A, 18008 Granada, Spain

**Keywords:** Authors’ editor, Citation, Core competencies, Decision-making editor, English language, Journal policies, Plagiarism, Scientific editor

## Abstract

A team of stakeholders in biomedical publishing recently proposed a set of core competencies for journal editors, as a resource that can inform training programs for editors and ultimately improve the quality of the biomedical research literature. This initiative, still in its early stages, would benefit from additional sources of expert information. Based on our experiences as authors’ editors, we offer two suggestions on how to strengthen these competencies so that they better respond to the needs of readers and authors – the main users of and contributors to research journals. First, journal editors should be able to ensure that authors are given useful feedback on the language and writing in submitted manuscripts, beyond a (possibly incorrect) blanket judgement of whether the English is “acceptable” or not. Second, journal editors should be able to deal effectively with inappropriate text re-use and plagiarism. These additional competencies would, we believe, be valued by other stakeholders in biomedical research publication as markers of editorial quality.

## Correspondence

Journal editing cannot be learned in higher education, and alternative training opportunities are not readily available. To guide such training and ultimately improve the quality of published research, Moher and colleagues defined core competencies (CC) for editors of biomedical journals. They did a literature review ^1^, surveyed 148 journal editors ^2^, and used a Delphi-like process to rank different competencies ^2^, resulting in a consensus statement signed by 30 stakeholders in research publishing ^3^. We commend this initiative to help journal editors work responsibly and accountably, and offer suggestions on areas that might benefit from additional input.

Moher *et al.* do not use the term “journal editor” but rather “scientific editor”, defined as someone “who make[s] decisions on the content and policies of a journal — including editors-in-chief and associate/academic editors” ^2^. This definition excludes other editors who contribute to the quality of research publications, in contrast to the broader meaning of “science editor” used by two stakeholder groups in the consensus initiative (Council of Science Editors, European Association of Science Editors). To avoid confusion regarding who the CC are intended for, a term such as “decision-making editor” might be helpful. For simplicity’s sake, here we use “journal editor” to refer to the type of editor we assume the CC are intended for.

As Moher *et al.* concede, “time and resource constraints … limited inclusion of perspectives of other relevant groups (e.g. authors, readers, peer reviewers)” in developing the CC ^3^. We believe input from authors is fundamental to efforts to define competencies of journal editors, and suggest that insights into authors’ (sometimes less than satisfactory) experiences with journals can be provided by another type of editor, namely authors’ editors ^4– 10^. These editors help researcher-authors prepare manuscripts for publication by reading drafts and suggesting changes to structure and content (substantive editing), language and style (language editing), and appearance and format (e.g. compliance with journals’ instructions) ^9, 11^. In addition, many authors’ editors train researchers in publication skills ^12– 17^ and help authors navigate editorial processes ^18– 21^. Authors’ editors’ knowledge of the publication process and their close interactions with the producers, distributors and consumers of research information make them qualified to help define CC for journal editors and identify deficiencies in current practices ^8^.

Authors’ editors are often more familiar with researchers’ local circumstances and challenges than journal editors are. Although the writers of the consensus statement and their informants are themselves researchers and therefore authors, they were perhaps not representative of the wider population of “real-world” researchers who have limited contact with English-speaking opinion leaders in biomedical publishing. In contrast, many authors’ editors work with researchers whose first language is not English or who are based outside the global North and West. Familiarity with other languages and cultures gives authors’ editors insights into the types of competencies researchers from diverse geographical, cultural and linguistic backgrounds would value in journal editors.

Like journal editors, authors’ editors are taking steps to critically evaluate and improve their working methods, considering that, for this profession too, there is no single agreed-upon academic degree or CC-inspired training program. Among these efforts, a growing body of literature ^9^ facilitates knowledge transfer to colleagues in different settings. PhD degrees have been awarded to authors’ editors for applied linguistics research based on their work practices in the Netherlands ^22^, Spain ^23^ and China (Luo ^24^ and unpublished; available upon request). Continuing professional development for authors’ editors is available through national and international associations ( Table 1). Authors’ editors in these associations can provide valuable information about journal editor CC that researchers would value. As authors’ editors ourselves, we offer suggestions on how to improve the CC based on insights we and our colleagues gain about researchers’ experiences with peer review.

**Table 1.** Professional associations that provide continuing professional development for authors’ editors (and other types of editors).

Association	Membership ^a^	Year founded	Website
Editors’ Association of Canada (Editors Canada)	Canada and North America	1979	www.editors.ca
Society of Writers, Editors and Translators (SWET)	Japan	1981	www.swet.jp
Society for Editors and Proofreaders (SfEP)	UK and Europe	1988	www.sfep.org.uk
Society of English-language professionals in the Netherlands (SENSE)	The Netherlands and Europe	1990	www.sense-online.nl
Institute of Professional Editors (IPEd)	Australia	1998	www.iped-editors.org
Asociación Española de Traductores, Correctores e Intérpretes (Asetrad)	Spain and Europe	2003	www.asetrad.org
Mediterranean Editors and Translators (MET)	Spain and Europe	2006	www.metmeetings.org
Nordic Editors and Translators (NEaT)	Finland and Europe	2014	nordicedit.fi

^a^ Country and continent in which most members live

In our experience, reviewers and journal editors increasingly cite “problems with the English” as a reason for rejection, even of manuscripts free from language errors. Meanwhile, biomedical journals publish an ever-increasing proportion of articles that were judged by reviewers to have “acceptable English” but which contain awkwardly worded statements that defy comprehension and undermine reproducibility ^9(pp5–9)^. To avoid these problems, journal editors should be able to either provide authors with useful feedback on the language (e.g. by endorsing or overruling reviewers’ complaints) or delegate this responsibility to an appropriately skilled reviewer or editorial staffer. Relying solely on blanket “acceptable/unacceptable” assessments of the writing contributes, in our experience, to cynicism among authors regarding the fairness and quality of peer review, and to the proliferation of poorly written articles. Although we realize that skills in “dealing with language issues” and knowledge about the “fundamentals of editing” were considered but then excluded from the CC ^2^, we believe that inclusion of a competency in this area would be welcomed and perceived as a marker of editorial quality. To start with, journal editors should have a basic understanding of different types of editing services, including those proposed by their own journals, so that they can refer authors to a suitable service whenever necessary.

Another competency researchers would appreciate is the ability of journal editors to deal effectively with inappropriate text re-use. This omission from the CC is surprising, especially since plagiarism featured in two of the 23 highly ranked statements in the Delphi process ^2^. While working with authors on manuscripts, authors’ editors sometimes encounter re-used text and inadequate citation, and use these opportunities to explain why these practices may be inappropriate and how to avoid them ^25^. But these individual efforts are not enough to stop the global spread of plagiarism in published research, which journal editors may inadvertently facilitate if they do not check manuscripts carefully enough before publication. Journal editors should be able to interpret the results of “plagiarism-detection” software and deal sensitively with the manuscripts these tools single out (as proposed by the Committee on Publication Ethics, publicationethics.org/files/u2/02A_Plagiarism_Submitted.pdf). Setting a maximum allowable percentage of text overlap, without considering the context of the non-original text, may send inconsistent messages about appropriate and inappropriate text re-use. Manuscript rejection based solely on the percentage of non-original text can, in our experience, alienate well-meaning authors from journals that use this criterion.

These are just two of the areas where authors’ editors can provide valuable input for future efforts to define and refine CC for biomedical journal editors. Alongside earlier efforts to support professional and ethical practices ^26– 28^ (see also: publicationethics.org/files/editable-bean/COPE_Core_Practices_0.pdf, and www.wame.org/about/syllabus-for-prospective-and-newly-appointed), the CC may indeed help gatekeepers meet researchers’ and readers’ expectations for editorial practices that ultimately improve the quality of published research.
