# Supplementary material for: Improving the biomedical research literature: insights from authors’ editors can help journal editors define and refine their core competencies
Source: F1000Res. 2018 Feb 21;7:109. Originally published 2018 Jan 25. [Version 2] doi: 10.12688/f1000research.13760.2 (PMC5811667; doi:10.12688/f1000research.13760.2)
Supplement: Peer review history

# Peer review: Referee response for version 1

**DOI:** 10.5256/f1000research.14953.r30328

**Published:** 2018-02-12

**Authors:** Na Luo

**Affiliations:** 1 School of Foreign Languages, Wuhan University of Science and Technology, Wuhan, China

In this correspondence, two well-known European authors’ editors, Dr. Valerie Matarese and Ms. Karen Shashok, raise two very interesting points to enhance the core competencies (CC) list for biomed journal editors in Moher *et al* (2017).  They aptly argue that the perspective from authors is essential to define journal editor CC and thus inappropriate to be overlooked. Then they indicate that authors’ editors can give insights on authors’ behalf when reliable author opinion is not readily available by calling attention to the work of authors’ editors and their advantage over journal editors in familiarity with authors in different contexts.

 The two points they suggest to be added to the CC list of Moher *et al* (2017) are not only insightful but also very caring for most EAL (English as an additional language) scholars who often struggle to publish in a language they do not have much control. The first one is that the CC list should re-include journal editors’ competence to give useful feedback on language and writing to authors instead of relying on standardized reviewer reports where this aspect is simply judged “acceptable” or “unacceptable”. In a highly globalized academic community where biomedical journals are at the very forefront of globalization, it is unrealistic to expect most reviewers to give concrete language and writing feedback to researchers, particularly EAL ones. Therefore, it seems incumbent on journal editors to possess this as a core competence. What I am thinking here is that journal editors may also need more knowledge of authors’ editors. This is because EAL researchers need to be referred to the right authors’ editors for support when fixing the language and writing problems goes beyond their own capacity.

 The second competence Matarese and Shashok suggest to add is that journal editors should be able to interpret results of plagiarism-detection software sensibly and sensitively rather than simplistically relying on arbitrarily set maximum-text-overlap percentages. As is known to writing researchers and authors’ editors, text re-use is an important, if not the most, way for EAL scholars to learn academic writing and compose papers in English. Journal editors’ lack of ability to tell the difference between plagiarism and well-meant text re-use can render them to punish the innocent and alienate EAL scholars from their journals, as convincingly argued by the two authors of this piece.

 Overall, I find the suggestions of Matarese and Shashok very useful to refine the CC list for journal editors, as given in Moher *et al *(2017), in a well-crafted text. Their effort to support EAL scholars in ways beyond working on manuscripts should be commended. Therefore, I recommend that this correspondence be indexed as soon as possible. If they would like to add that journal editors also need to have the ability to refer EAL scholars to proper authors’ editors, I would appreciate this piece even more.

I have read this submission. I believe that I have an appropriate level of expertise to confirm that it is of an acceptable scientific standard.

# Response

**Published:** 2018-02-19

**Authors:** Karen Shashok

**Affiliations:** Translator - Editorial consultant, Spain

We concur with Dr. Luo’s understanding of language and writing in the current globalized context of research publishing, including some EAL scholars’ recourse to text re-use to produce acceptable English. We also agree that suggestions by journal editors on where authors could obtain help is a competency that researchers are likely to value, and have noted this in version 2. Many journals already list commercial editing service providers on their websites, but the scope and quality of these services vary.

 Journal editors have differing criteria for “acceptable” English or writing, but as the ultimate arbiters of when the language and writing are “good enough” for publication in their journal, they should, for the sake of transparency and fairness, let authors know what criteria they use to decide which texts are publishable and which need improvement.

# Peer review: Referee response for version 1

**DOI:** 10.5256/f1000research.14953.r30325

**Published:** 2018-02-05

**Authors:** Joy Burrough-Boenisch

**Affiliations:** 1 Unclogged English, Renkum, Netherlands

Written by two long-standing, respected professional biomedical authors’ editors, this article responds to the proposal to create core competencies (CC) “for scientific editors of biomedical journals”. Although commending the proposal, Matarese and Shashok point out that Moher *et al.’*s use of the term “scientific editor”, instead of “journal editor” or “decision-making editor", might raise confusion about the type of editor to which the CC apply. They also argue that when refining the CC, authors’ experiences with journals provide useful insights into the CC needed by journal editors.

 Matarese and Shashok point out that some scientific editors do not work for a publisher or journal but instead work closely with researcher-authors, helping them to produce publishable papers – a service particularly helpful to authors writing in their second (or third) language or living in the Global South. These “authors’ editors” can also help their author clients to deal with reviewer comments and journal-related correspondence, thereby accumulating valuable information on authors’ experiences with journal editors. Authors’ editors are thus well-placed to contribute to formulating certain CC, especially in relation to assessing the quality of the English and providing clear, constructive feedback to authors. Drawing on their extensive knowledge of journal–author intercommunication, Matarese and Shashok plead for reversal of the decision not to include competence to deal with language issues and knowledge of the “fundamentals of editing” from the CC. They also express surprise at another omission: competency in plagiarism detection. Again, they point out relevant insights that authors’ editors can supply.

 Matarese and Shashok’s case for expanding and refining the CC with the help of authors’ editors is convincingly argued. They have underpinned it with references to relevant publications by authors’ editors that are probably unfamiliar to editors of science journals. The table of editors’ associations indicates that there are professional associations providing CPD that committed, principled authors’ editors may join. All this helps broadcast the actual and potential usefulness of authors’ editors to researcher-authors, journals, journal reviewers and journal editors. However, to avoid creating the impression that all authors’ editors are suitably trained, optimally competent in all the editing they undertake, involved members of appropriate associations and proactive in undertaking CPD, Matarese and Shashok should acknowledge that there are no CC for authors’ editors, nor are authors’ editors required to have certain academic or professional qualifications. Matarese and Shashok are currently among the most respected and high-profile biomedical authors’ editors; their plea to involve authors’ editors in developing CC for journal editors should be placed in that context.

I have read this submission. I believe that I have an appropriate level of expertise to confirm that it is of an acceptable scientific standard.

# Response

**Published:** 2018-02-19

**Authors:** Karen Shashok

**Affiliations:** Translator - Editorial consultant, Spain

We appreciate Dr. Burrough-Boenisch’s cautionary note on the variations in authors’ editors’ skills, practices and commitment to continuing education, which are to be expected for any profession that does not have defined entry qualifications or agreed-upon CC.

 The author editing literature contains several proposals for key skills and tasks (see, for example, Lang 1999; Burrough-Boenisch 2013; Chapter 7 in Matarese 2016), but although they overlap to some extent, they also tend to reflect the particular circumstances of different authors’ editors’ professional settings, clients and disciplinary focus. Although several professional associations offer formal certification, these programs do not cover all facets of author editing, such as the interpersonal aspects of working with researchers and the value of multilingualism. Like journal editors, authors’ editors with different skills, strengths and specialist knowledge can provide valuable services in some areas, but may have shortcomings in other areas. Our impression is that authors’ editors are likely to be transparent about their strengths and limitations, in order to help researchers decide whether they are able to provide the right types of support.

 We have noted the lack of universally agreed-upon professional qualifications for authors’ editors in version 2.

 Burrough-Boenisch J: Editing non-native English: reflections from a Netherlands-based editor on those who do it and the skills they should have. The 21st Century Text. July 30, 2013.

 Lang TA: A curriculum for biomedical writing and editing: a second volley. CBE Views. 1999; 22(1): 3–5.

 Matarese V: Editing research: the author editing approach to providing effective support to writers of research papers. Medford: Information Today; 2016.
